# Supplementary material for: Citizens’ feedback on health service and the responses of health authorities of Bangladesh: An analysis of the Grievance Redress System
Source: PLOS Digit Health. 2025 Jul 30;4(7):e0000967. doi: 10.1371/journal.pdig.0000967 (PMC12310018; doi:10.1371/journal.pdig.0000967)
Supplement: S1 File — (DOCX) [file pdig.0000967.s001.docx]

**
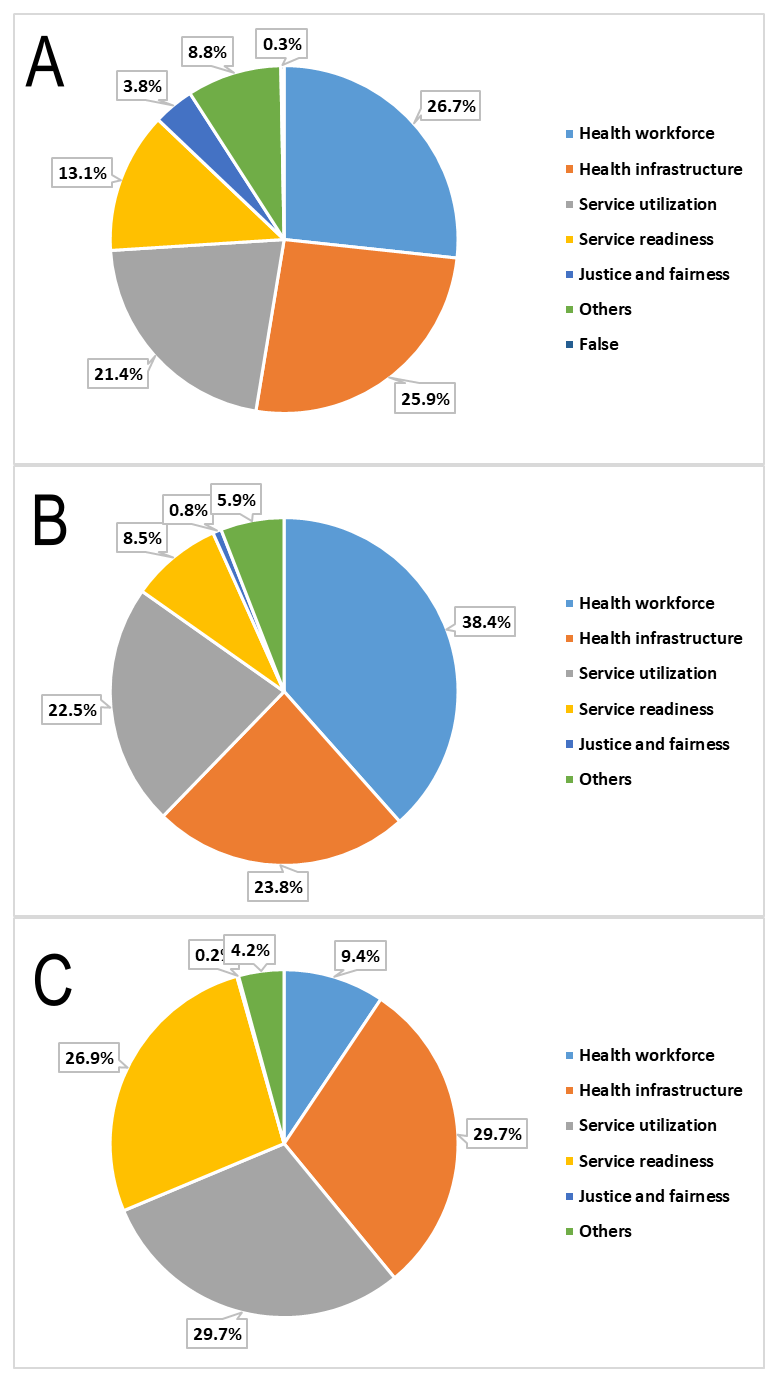
Fig A. Category-wise distribution of complaints (A), suggestions (B) and Compliments (C)**

**Fig B. Distribution of health workforce-related complaints**

**Fig C. Distribution of health infrastructure-related complaints**

**Fig D. Distribution of justice and fairness-related complaints**

**Fig E. Distribution of service readiness-related complaints**

**Fig F. Distribution of service utilization-related complaints**
