# Supplementary material for: Citizens’ feedback on health service and the responses of health authorities of Bangladesh: An analysis of the Grievance Redress System
Source: PLOS Digit Health. 2025 Jul 30;4(7):e0000967. doi: 10.1371/journal.pdig.0000967 (PMC12310018; doi:10.1371/journal.pdig.0000967)
Supplement: S1 Table — (DOCX) [file pdig.0000967.s002.docx]

**S1 Table. Division wise distribution of feedback type**

| **Division** | **Complaint** | **Compliment** | **Suggestion** | **Total** |
| --- | --- | --- | --- | --- |
| Barisal | 315 (4.92) | 125 (5.33) | 52 (3.35) | 492 (4.78) |
| Chattogram | 1,229 (19.22) | 429 (18.28) | 263 (16.92) | 1,921 (18.66) |
| Dhaka | 1,164 (18.20) | 341 (14.53) | 216 (13.90) | 1,721 (16.71) |
| Khulna | 969 (15.15) | 532 (22.67) | 244 (15.70) | 1,745 (16.95) |
| Mymensingh | 518 (8.10) | 250 (10.65) | 139 (8.94) | 907 (8.81) |
| Rajshahi | 1,336 (20.89) | 473 (20.15) | 502 (32.30) | 2,311 (22.44) |
| Rangpur | 456 (7.13) | 124 (5.28) | 109 (7.01) | 689 (6.69) |
| Sylhet | 409 (6.39) | 73 (3.11) | 29 (1.87) | 511 (4.96) |

Note: Data within parenthesis represents column percentage.
