# Supplementary material for: Citizens’ feedback on health service and the responses of health authorities of Bangladesh: An analysis of the Grievance Redress System
Source: PLOS Digit Health. 2025 Jul 30;4(7):e0000967. doi: 10.1371/journal.pdig.0000967 (PMC12310018; doi:10.1371/journal.pdig.0000967)
Supplement: Attachment — Submitted filename: Response to reviewers.docx [file pdig.0000967.s004.docx]

Dear Editor,

First of all, we want to express our sincere gratitude to the editors and reviewers for their time and valuable feedback. We believe the manuscript has substantially improved after the adjustments to the reviews. Herein, we present a point-by-point response to the comments of the editors and reviewers. Each answer is placed immediately below the comment.

Best regards,
Md Abdullah Saeed Khan

Corresponding author

**Journal Requirements:**
1. Please provide an Author Summary. This should appear in your manuscript between the Abstract (if applicable) and the Introduction, and should be 150–200 words long. The aim should be to make your findings accessible to a wide audience that includes both scientists and non-scientists. Sample summaries can be found on our website under Submission Guidelines
[https://journals.plos.org/digitalhealth/s/submission-guidelines#loc-parts-of-a-submission](https://track.editorialmanager.com/CL0/https:%2F%2Fjournals.plos.org%2Fdigitalhealth%2Fs%2Fsubmission-guidelines%23loc-parts-of-a-submission/1/010f0196ef2e0844-2e73ea3c-0ead-492e-881c-d093f4c14a88-000000/TK0KnWW0vTv3ClXC0nOacm0GoYtEhpvTQUImHoXXmQE=212)

**Authors’ response:** Thank you for the advice. We added an author summary section in between the abstract and introduction sections: lines 44 to 58.

2. Figure 1: please (a) provide a direct link to the base layer of the map (i.e., the country or region border shape) and ensure this is also included in the figure legend; and (b) provide a link to the terms of use / license information for the base layer image or shapefile. We cannot publish proprietary or copyrighted maps (e.g. Google Maps, Mapquest) and the terms of use for your map base layer must be compatible with our CC-BY 4.0 license.

Note: if you created the map in a software program like R or ArcGIS, please locate and indicate the source of the basemap shapefile onto which data has been plotted.

If your map was obtained from a copyrighted source please amend the figure so that the base map used is from an openly available source. Alternatively, please provide explicit written permission from the copyright holder granting you the right to publish the material under our CC-BY 4.0 license.

Please note that the following CC BY licenses are compatible with PLOS license: CC BY 4.0, CC BY 2.0 and CC BY 3.0, meanwhile such licenses as CC BY-ND 3.0 and others are not compatible due to additional restrictions.

If you are unsure whether you can use a map or not, please do reach out and we will be able to help you. The following websites are good examples of where you can source open access or public domain maps:
* U.S. Geological Survey (USGS) - All maps are in the public domain. (<http://www.usgs.gov>)
* PlaniGlobe - All maps are published under a Creative Commons license so please cite “PlaniGlobe, <http://www.planiglobe.com>, CC BY 2.0” in the image credit after the caption. (<http://www.planiglobe.com/?lang=enl>)
* Natural Earth - All maps are public domain. (<http://www.naturalearthdata.com/about/terms-of-use/>)

**Authors’ response:** Thank you again for the suggestion. We added the link to the source of the shapefile used to create the map using ArcGIS with the type of license [CC BY 4.0] in the figure legend.

**Additional Editor Comments (if provided):**

This manuscript presents a large-scale analysis of feedback messages collected through the GRS, managed by the Ministry of Health and Family Welfare of Bangladesh. However, several critical issues must be addressed before the manuscript can be considered for publication.
1. The introduction extensively describes the context of globalization but does not adequately address the challenges facing Bangladesh’s health system. Although it concludes with 'we examined..., we explored...,' it fails to clearly articulate the research question, purpose, and significance of the study. It is recommended that the author include a clear statement explaining the study’s purpose and potential impact, such as its implications for public health systems, digital health platforms, or policymakers.

**Authors’ response:** Thank you for the suggestion. We modified the last part of the introduction as per the recommendation to clearly state the study objective and its implications for the practice and policy. See lines 96 to 105.

2. Page 5, lines 105–107: “Ranks include – major, moderate, and minor. These ranks are assigned by an expert of MIS based on the imminence of the complaints and suggestions, and the level of satisfaction determined from the language of the compliments.” The description of the classification criteria lacks clarity, potentially introducing a high degree of subjective bias. The author shall clarify whether the evaluation process incorporated mechanisms, such as a standardized operational manual, a dictionary, or a double-review process, to ensure consistency.

**Authors’ response:** Thank you for clarifying this. We improvised the description of the classification criteria based on the available information from a 2020 report on the GRS. Please check lines 135 to 149 for details.

3. Page 5, lines 109–114: The manuscript does not adequately define the closure type. What is the difference between 'Resolved' and 'Closed'? Is 'Forwarded' equivalent to 'Unprocessed'? Additionally, is there any further follow-up? The author should provide a more precise definition and explanation.

**Authors’ response:** Thank you for the suggestion. We modified the last part of the introduction as per the recommendation to clearly state the study objective and its implications for the practice and policy. See lines 96 to 105.

4. The study used Kaplan-Meier survival curves and log-rank tests, using 'resolution' as the event of interest. However, the resolution rate was low (2.55%). Please reassess or justify the appropriateness of survival analysis in this context, or consider adopting an alternative approach.

**Authors’ response:** Thank you for raising this interesting point. We went through the feedback types, rank, and closure type again. Considering the definitions of closures and nature feedback, we found that probably by nature of the messages, most of the suggestions are forwarded to relevant departments or ministries, and most of the compliments are, which do not contain points for actions to be taken on, closed. Therefore, comparing the resolution time across types of messages (Figure 2a) seems not to carry any relevant information. However, the second part of the figure (Figure 2b) compares the resolution time for different ranks of complaints. Here again, although most of the complaints are forwarded to other departments, the resolution is done by the officers involved with GRS and the Management Information System (MIS) of the Directorate General of Health Services, which occurs for all ranks of complaints. Overall, 2.16% (n=55), 3.69% (n=42), and 4.07% (n=131) of major, minor, and moderate **complaints** are resolved, respectively. As the KM Survival curve compares the time to resolution, it can give an idea of the probability of different types of complaints being resolved within a given period. So we kept this curve and removed Figure 2a. We have expanded the description of Figure 2b (now Figure 2) and the relevant discussion as well. Please check lines 211- 215 and 262 – 274.

5. Please expand the Discussion section (it is currently not there). The authors should consider including the following sections: implications for practice, implications for research, and implications for policy.

**Authors’ response:** Thank for the suggestion. The discussion section was there, which might have been missed because of the formatting. We have increased font size of the headings to improve readability. As per the suggestions, we expanded the discussion and added the recommended sections. Please check lines 289 – 308.

6. Please review the manuscript and correct grammatical errors.

**Authors’ response:** Thank you very much for the suggestion. We used Grammarly to check and correct the grammatical errors throughout the manuscript.

[Note: HTML markup is below. Please do not edit.]

**Reviewers' Comments:**

Reviewer's Responses to Questions

**Comments to the Author**

1. Does this manuscript meet PLOS Digital Health’s [publication criteria](https://track.editorialmanager.com/CL0/https:%2F%2Fjournals.plos.org%2Fdigitalhealth%2Fs%2Fjournal-information%23loc-criteria-for-publication/1/010f0196ef2e0844-2e73ea3c-0ead-492e-881c-d093f4c14a88-000000/lwu1bjtQTDAY-O4pmusGrD2tlhZBdN7u3kYZf4xZYN4=212)? Is the manuscript technically sound, and do the data support the conclusions? The manuscript must describe methodologically and ethically rigorous research with conclusions that are appropriately drawn based on the data presented.

Reviewer #1: Yes

Reviewer #2: Yes

**Authors’ response:** Thank you very much. We appreciate your effort and time to review the manuscript.

2. Has the statistical analysis been performed appropriately and rigorously?

Reviewer #1: Yes

Reviewer #2: Yes

**Authors’ response:** Thank you very much. We appreciate your effort and time to review the manuscript.

3. Have the authors made all data underlying the findings in their manuscript fully available (please refer to the Data Availability Statement at the start of the manuscript PDF file)?

The [PLOS Data policy](https://track.editorialmanager.com/CL0/https:%2F%2Fjournals.plos.org%2Fdigitalhealth%2Fs%2Fdata-availability/1/010f0196ef2e0844-2e73ea3c-0ead-492e-881c-d093f4c14a88-000000/HFIkfzMPCsvABk9_yLP6DU7ku2t5I8-9U5rA-gkx8qA=212) requires authors to make all data underlying the findings described in their manuscript fully available without restriction, with rare exception. The data should be provided as part of the manuscript or its supporting information, or deposited to a public repository. For example, in addition to summary statistics, the data points behind means, medians and variance measures should be available. If there are restrictions on publicly sharing data—e.g. participant privacy or use of data from a third party—those must be specified.

Reviewer #1: Yes

Reviewer #2: Yes

**Authors’ response:** Thank you very much. We appreciate your effort and time to review the manuscript.

4. Is the manuscript presented in an intelligible fashion and written in standard English?

PLOS Digital Health does not copyedit accepted manuscripts, so the language in submitted articles must be clear, correct, and unambiguous. Any typographical or grammatical errors should be corrected at revision, so please note any specific errors here.

Reviewer #1: Yes

Reviewer #2: Yes

**Authors’ response:** Thank you very much. We appreciate your effort and time to review the manuscript.

5. Review Comments to the Author

Please use the space provided to explain your answers to the questions above. You may also include additional comments for the author, including concerns about dual publication, research ethics, or publication ethics. (Please upload your review as an attachment if it exceeds 20,000 characters)

Reviewer #1: 1. Abbreviations must use at first place, multiple place this flaws found.

**Authors’ response:** Thank you very much. We corrected these throughout the manuscript. We only gave the abbreviations in their first instances.

2. Provide examples of complaints, suggestions, and compliments to help contextualize the categories. For example:
Complaints: "Long wait times, lack of doctors, or inadequate facilities."
Suggestions: "Recommendations to improve appointment scheduling systems."

**Authors’ response:** Thank you very much for the suggestions. We added some examples, including your suggestions (after cross-checking the message content in the original data), in the method section to help contextualize the categories. Please check lines 130 to 132.

3. The percentages for response types (e.g., forwarding, closure, resolution) are useful, but a short explanation about why "resolution" is low (2.55%) could add value. For instance, are there systemic bottlenecks preventing resolution?

**Authors’ response:** Thank you very much for the suggestions. We have expanded the discussion for the low resolution rate. Please check lines 260-279.

Reviewer #2: This is an important report to help keep the quality of services front of mind in Bangladesh. One question I had that might be included, does the geographic distribution of complaints relate in anyway to low resourced vs. high resourced areas?

**Authors’ response:** Thank you for raising this interesting question. We can expect the division district should have high resources. However, if we carefully look at the map in Figure 1, we can see that the distribution of complaints did not follow such a pattern. Because a high number of complaints came from Rajshahi and Khulna districts. Whereas low frequency of complaints came from other divisional districts. Moreover, complaints were very low from hilly areas, boundary districts, as well as peri-capital regions.

6. PLOS authors have the option to publish the peer review history of their article ([what does this mean?](https://track.editorialmanager.com/CL0/https:%2F%2Fjournals.plos.org%2Fdigitalhealth%2Fs%2Feditorial-and-peer-review-process%23loc-peer-review-history/1/010f0196ef2e0844-2e73ea3c-0ead-492e-881c-d093f4c14a88-000000/k8jZyMOduGHMwbwf71xH2JPIt2ocYMz38RQJWrFV82E=212)). If published, this will include your full peer review and any attached files.


**Do you want your identity to be public for this peer review?** If you choose “no”, your identity will remain anonymous but your review may still be made public.


For information about this choice, including consent withdrawal, please see our [Privacy Policy](https://track.editorialmanager.com/CL0/https:%2F%2Fwww.plos.org%2Fprivacy-policy/1/010f0196ef2e0844-2e73ea3c-0ead-492e-881c-d093f4c14a88-000000/OcrLqE9u_KzLKis9E0rbo6LIXqwhfRehIwhS1iWYsDU=212).

Reviewer #1: No

Reviewer #2: No
